# Analysis of Misbehaviors and Satisfaction With School in Secondary Education According to Student Gender and Teaching Competence

**DOI:** 10.3389/fpsyg.2020.00063

**Published:** 2020-01-28

**Authors:** Antonio Granero-Gallegos, Raúl Baños, Antonio Baena-Extremera, Marina Martínez-Molina

**Affiliations:** ^1^Faculty of Education Sciences, University of Almería, Almería, Spain; ^2^Health Research Center, University of Almería, Almería, Spain; ^3^Department of Physical Education and Sports Science, Autonomous University of Baja California, Ensenada, Mexico; ^4^Department of Didactic of Corporal Expression, Faculty of Education Sciences, Granada, Spain

**Keywords:** physical education, disruptive behavior, indiscipline, high school, satisfaction

## Abstract

Effective classroom management is a critical teaching skill and a key concern for educators. Disruptive behaviors disturb effective classroom management and can influence school satisfaction if the teacher does not have the competencies to control them. Two objectives were set in this work: to understand the differences that exist in school satisfaction, disruptive behaviors, and teaching competencies according to the gender of the students; and to analyze school satisfaction and disruptive student behaviors based on perceived teaching competence. A non-probabilistic and convenience sample selection process was employed, based on the subjects that we were able to access. 758 students participated (male = 45.8%) from seven public secondary schools in the Murcia Region (Spain). The age range was between 13 and 18 years (*M* = 15.22; *DT* = 1.27). A questionnaire composed of the following scales was used: *Competencies Evaluation Scale for Teachers in Physical Education, School Satisfaction* and *Disruptive Behaviors in Physical Education.* Mixed Linear Models performed with the SPSS v.23 was used for statistical analyses. The results revealed statistically significant differences based on gender and physical education teaching competencies. In conclusion, the study highlights that physical education teacher skills influence disruptive behaviors in the classroom, and that this is also related to school satisfaction. Furthermore, it highlights that boys showed higher levels of negative behaviors than girls.

## Introduction

Undisciplined behaviors in the classroom are a serious problem for the teaching and learning process during adolescence (Medina and Reverte, 2019), and may have an impact on feelings regarding school satisfaction, the relationship with teachers or even on school failure (Baños et al., 2017). These types of behaviors commonly occur in the Physical Education (PE) class, producing conflictive situations between peers (students) and even with the teacher himself/herself. It is therefore advisable to solve the problem in a rapid and effective fashion (Müller et al., 2018). Faced with these situations, the competencies of the PE teaching staff play an important role (Baños et al., 2017; Trigueros and Navarro, 2019; Granero-Gallegos et al., 2020); the way in which teachers design, organize and control their sessions can affect the students’ disruptive behaviors and class outcome.

As evidenced in research by authors such as Goyette et al. (2000) and Kulinna et al. (2006), adolescents often show certain problematic behaviors in the classroom, such as idleness, disrespect, talking out of turn and/or avoiding or skipping classes, which have a negative impact on the learning environment. Even aggressive behaviors can sometimes arise in PE classes, such as bullying and peer fighting (Weiss et al., 2008). Studies looking at inappropriate behaviors in PE have demonstrated that the students’ negative behavior not only affects the quality of teaching, but also interferes with peer learning (Kulinna et al., 2006; Cothran et al., 2009). Moreover, disruptive behaviors are more common at the secondary school level than in primary education classes, as evidenced by various works (e.g., Kulinna et al., 2006; Cothran and Kulinna, 2007). Adolescence, in particular, is characterized by a rebellious, non-conformist stage, a fight against authority, irresponsibility and low personal self-control. At this age, a disengagement with the school can occur, with a decreased willingness to comply with the rules and with expected behavior (Fredericks et al., 2004).

In addition, gender has been used to analyze these behaviors, both in students and in teachers. Specifically, the female gender (both teachers and students) are those who report the highest incidence of inappropriate behaviors (Kulinna et al., 2006), with females being the ones likely to receive this negativity (Cothran and Kulinna, 2007). There are several studies that have found higher levels of inappropriate behaviors among boys than among girls (Beaman et al., 2006; Kulinna et al., 2006; Cothran and Kulinna, 2007; Driessen, 2011). Boys tend to be more boisterous and disruptive with their peers (Glock and Kleen, 2017) whereas girls tend to be more proactive and less problematic (Driessen, 2011), albeit with more shy and introverted behaviors (Glock and Kleen, 2017). Furthermore, boys are often more influenced by their peers than girls are, resulting in higher levels of truancy, punishments and challenging behaviors that teachers have to face (Hadjar and Buchmann, 2016; Geven et al., 2017). Other authors (i.e., Baños et al., 2018) found that students claimed to have more aggressive behaviors during PE sessions.

Among the attributions made by the students regarding inappropriate behaviors when doing PE, the boredom they experience stands out, finding the classes monotonous, as well as expressing a certain discontent with the teacher. However, it should be noted that these are students with usually disruptive behaviors (Cothran and Kulinna, 2007). In relation to the teachers, some recent studies have linked disruptive behaviors to teacher competence as perceived by the students (Baños et al., 2019; Granero-Gallegos et al., 2019; Granero-Gallegos et al., 2020). This research related the high levels of teaching competencies with low levels of negative behavior in PE classes although the study did not cover the effect of the teaching staff’s competence.

In addition, the scientific literature has stated that school satisfaction reduces student misbehavior, making it advisable to develop social and emotional skills, cognitive ability, behavioral and moral competencies, the recognition of positive behavior, belief in the future and prosocial norms (Sun, 2016). In contrast, ineffective classroom management causes disarray and interruptions produced by a few adolescents, affecting both the anxiety and stress of their peers and that of the teachers (Cothran et al., 2009).

In this way, the work of PE teachers plays a relevant part in developing good classroom behaviors. Depending on the skills that the teachers develop, they may increase or decrease negative behaviors (Rasmussen et al., 2014). Thus, teachers who have a wide repertoire of teaching styles, and who know how to adapt them to different environments and learning content, manage to improve the students’ satisfaction with the school (Invernizzi et al., 2019); this is also influenced by the orientations toward learning (Agbuga et al., 2010).

Regarding the study of satisfaction, Diener’s theory of subjective well-being (Diener, 2009) could be of great help. This theory consists of two dimensions, the cognitive dimension and the affective dimension. The cognitive dimension relates to the evaluative judgments of global satisfaction with life and its specific areas, while the affective dimension is identified with emotions and attachments such as fun, boredom and concern (Diener and Emmons, 1985). In this vein, Baena-Extremera and Granero-Gallegos (2015) highlight the importance of the student being satisfied and at ease in school. An adolescent who is satisfied with the school is associated with higher levels of life satisfaction (Scharenberg, 2016), with an adequate school climate managed by the teacher (Varela et al., 2018) and with better social relationships among his/her peers (Persson et al., 2016). However, a student who gets bored at school decreases the efficiency of any learning style (Ahmed et al., 2013). This is associated with higher school dropout rates (Takakura et al., 2010), and with low teacher competencies (Sun, 2016), which in turn relates to greater disruptive behavior (Baños et al., 2019).

Scientific evidence has demonstrated the impact of negative behaviors and student satisfaction on both the learning and teaching processes. However, there is not enough literature that links the skills of the PE teacher with either student satisfaction with the school or with classroom misbehavior. Therefore, this work sets out two important objectives: (1) to understand the differences that exist in terms of school satisfaction, disruptive behaviors and teaching competencies according to the gender of the students; and (2) to analyze school satisfaction and disruptive student behaviors based on perceived teaching competence. From a review of the literature, the following hypotheses are made:

(1) There will be a significant and positive correlation between school satisfaction, disruptive student behaviors and the perceived competencies of the PE teacher; however, there will be a significant and negative correlation between boredom with school, disruptive student behaviors, and the perceived competencies of the PE teacher.
(2) Boys will show more negative behaviors than girls although girls will score higher in school satisfaction and in the perception of teaching competencies.
(3) Students who perceive that PE teachers are competent will show less disruptive behavior and greater school satisfaction.

## Materials and Methods

### Participants

The design of this cross-sectional study was observational and descriptive selecting a non-probabilistic convenience sample according to the people that could be accessed from public high schools located in areas of medium socioeconomic level (from Murcia and Cartagena cities). No educational center is included in the program of Teaching Compensatory, program that allocates specific, material and human resources to guarantee access, permanence and promotion in the educational system for socially disadvantaged students. A total of 758 students participated (males = 45.8%) from seven public secondary schools in the Murcia region of Spain (94% Spanish, Caucasian; 4% Arab origin; 1% East European, Caucasian; 1% South American). All students of these educational centers from 2^nd^, 3^rd^, 4^th^ of ESO and 1^st^ of Baccalaureate (PE is also subject compulsory) were requested to participate in this research. Incomplete answers due to errors or omissions in their responses (28) were dismissed for analysis and 34 students did not obtain parental consent to participate in this investigation. The age range was between 13 and 18 years (*M* = 15.22; *SD* = 1.27); the average age for the boys was 15.2 (*SD* = 1.29) and for the girls was 15.18 (*SD* = 1.26). The distribution in terms of course levels was as follows: 45.3% at ESO 2^nd^ level; 20.1% at ESO 3^rd^ level; 27.2% at ESO 4^th^ level; and 7.5% in the 1^st^ year of Baccalaureate. As PE is a compulsory subject for all students of the 1^st^ year of Baccalaureate, these students were also included in this research. There were no statistically significant differences in gender × age between the included participants (*p* = 0.501) (see Table 1).

**TABLE 1:** Distribution of the sample (n) according to Gender × Age (*p* = 0.501).

		Age			
		
		13 and 14 years	15 and 16 years	17 and 18 years	Total
Gender	Girl	210	164	37	411
	Boys	166	153	28	347
	Total	376	317	65	758

### Instruments

To carry out this investigation, the next instruments have been used.

#### Teaching Competence

The Spanish version of the *Competencies Evaluation Scale for Teachers in Physical Education* (ETCS-PE) by Baena-Extremera et al. (2015) was used, adapted from the original *Evaluation of Teaching Competencies Scale* by Catano and Harvey (2011). It consists of eight items that measure the students’ perception of teacher effectiveness. A seven-point Likert scale ranging from *low* (1, 2), *medium* (3, 4, 5), and *high* (6, 7) was used for the responses. The internal consistency indices were: Cronbach alpha (α) = 0.86; composite reliability = 0.86; Average Variance Extracted (AVE) = 0.59.

#### School Satisfaction

The Spanish version of the *Intrinsic Satisfaction Classroom Questionnaire* (ISC) by Castillo et al. (2001) was used, adapted from the original *Intrinsic Satisfaction Classroom Scale* by Nicholls et al. (1985), Nicholls (1989), and Duda and Nicholls (1992). It consists of eight items that measure the degree of school satisfaction using two subscales that measure *satisfaction/fun* (five items) and *boredom with school* (three items). For the responses, a Likert scale ranging from 1 (*totally disagree*) and 5 (*totally agree*) was used. The internal consistency indices were: *satisfaction/fun* α = 0.76, composite reliability = 0.76, AVE = 0.54; *boredom*, α = 0.70; Composite reliability = 0.72; AVE = 0.52.

#### Disruptive Behaviors in Physical Education

The Disruptive Conduct in Physical Education Questionnaire (CCDEF) by Granero-Gallegos and Baena-Extremera (2016) was used, which is the Spanish version of the original *Physical Education Classroom Instrument* (PECI) by Krech et al. (2010). This version consists of 17 items that measure disruptive behaviors in PE students in five subscales: (a) *Aggressive* (2 items), (b) *Low engagement or irresponsibility* (4 items), (c) *Fails to follow directions* (4 items), (d) *Distracts or disturbs others* (4 items), and (e) *Poor self-management* (3 items). A five-point Likert scale ranging from 1 (*never) to 5* (*always)* was used for the responses. The internal consistency indices were: *aggressive*,α = 0.58, composite reliability = 0.81, AVE = 0.54; *low engagement or irresponsibility*, α = 0.73, composite reliability = 0.84, AVE = 0.74; *fails to follow directions*, α = 0.77, composite reliability = 0.94, AVE = 0.65; *distracts or disturbs others*, α = 0.81, composite reliability = 0.92, AVE = 0.80; *poor self-management*, α = 0.84, composite reliability = 0.96, AVE = 0.92. Given the low index achieved by Cronbach’s alpha, and that the AGR subscale consists of only two items, this factor was ignored in the analyses performed.

### Procedure

Permission to carry out the work was obtained from the competent bodies, be they at the secondary schools or the university. Parents and adolescents were informed about the protocol and the study’s subject matter. Informed consent by both was an indispensable requirement to participate in the research. The tools measuring the different variables were administered in the classroom by the researchers themselves, without the teacher present. All participants were informed of the study objective, the voluntary and confidential nature of the responses and the data handling, as well as their rights as participants under the Helsinki Declaration (2008). This research has been approved by the Ethics Committee of the University of Murcia (REF-45-20/01/2016).

The questionnaires where completed in the classroom in about 25–30 min with the same researcher always present who expressed the possibility of consulting him about any doubts during the process, respecting the Helsinki Declaration (2008).

### Data Analysis

The descriptive statistics of the items, the correlations and the internal consistency of each subscale were calculated, as well as the asymmetry and kurtosis with values close to 0 and <2.0. It is important to note that the data from this work were collected in schools so that the students could be nested based on the center, course and/or class, that is, violating the independence of observations principle. Therefore, the Mixed Linear Models analysis (MLM) were conducted, bearing in mind the individual characteristic variables of the participants and context variables. The dependent variables were the different ETCS-PE, ISC and CCDEF subscales, and the grouping or level of the school was considered a random effect, as were the student courses. The analyses were performed using the SPSS 23.0 MIXED procedure with the Restricted Maximum Likelihood Estimation Method. The Logarithm of Likelihood -2 (-2LL) (Pardo et al., 2007) was used to estimate the effects of the school and course variable on each estimated model. Different models were tested according to the different combinations of school levels and course with each of the dependent variables, including a null model. The “school” variable proved statistically significant (*p* < 0.05) in all cases, so it was estimated that the context variable “school” had an effect on each model. In addition, the intraclass correlation coefficient (ICC) was calculated for each of the compared variables. The results showed that the variance explained was greater than 6.14% in all cases, which allows us to say that a percentage of the differences between the dependent variables can be attributed to the school. The estimation method used was the restricted maximum likelihood estimation method. In light of the above, gender differences in relation to the various ETCS-PE, ISC and CCDEF subscales were calculated, in this case, the independent variable (mixed model factors) was the gender of the students. To calculate the differences according to teaching competence, the responses of this scale were categorized into three groups, low (responses 1 and 2 on the Likert scale), medium (responses 3, 4, and 5) and high (responses 6 and 7). The calculation of the differences between the three categorized groups of teaching competence in relation to satisfaction and boredom with school and disruptive behaviors was also conducted and, in this case, the independent variable (mixed model factors) was the teaching competence categorization.

Additionally, the factorial structure of each instrument was evaluated with confirmatory factor analysis (CFA) using the Maximum Likelihood method with the bootstrapping procedure, since the Mardia coefficient was high in each of the scales (16.71 in ETCS-PE; 12.51 in ISC and 292.55 in CCDEF). The different analyses were performed using the SPSS v.23 and AMOS v.22 statistical packages.

#### Psychometric Properties of the Instruments

Based on recommendations that discourage the use of a single overall model-fit measure (Bentler, 2007), each model was assessed using a combination of absolute and relative fit indices. The chi-squared ratio (χ^2^) and the degrees of freedom (df) (χ^2^/df), the comparative fit index (CFI), the Tucker-Lewis index (TLI) the incremental fit index (IFI), the root mean square error of approximation (RMSEA) with its confidence interval (CI 90%) and the Standardized Root Mean Square Residual (SRMR) were calculated. In the (χ^2^/df) ratio, values < 2.0 are considered very good model fit indicators (Tabachnick and Fidell, 2007), although values < 5.0 are considered acceptable (Hu and Bentler, 1999). According to Hu and Bentler (1999), for the incremental indices (CFI, IFI, and TLI), values ≥ 0.95 are considered to indicate a good fit, although values of ≥ 0.90 are considered acceptable. These same authors consider that, for RMSEA, a value of ≤ 0.06 is considered to indicate a good fit, while for the RMSR values ≤ 0.08 are considered acceptable. As can be observed in Table 2, the different values for the goodness-of-fit indices of each instrument (ETCS-PE, ISC, and CCDEF) are acceptable.

**TABLE 2:** The goodness of fit index of the models.

	χ^2^	df	χ^2^/df	IFI	TLI	CFI	RMSEA(CI90%)	SRMR
ETCS-PE	36.04	20	1.80	0.99	0.99	0.99	0.03(0.01;0.05)	0.02
ISC	86.17	18	4.79	0.96	0.93	0.96	0.06(0.05;0.08)	0.04
CCDEF	303.09	84	3.61	0.96	0.95	0.96	0.06(0.05;0.07)	0.03

*χ^2^, chi-squared; df, degrees of freedom; IFI, incremental fit index; TLI, Tucker-Lewis index; CFI, comparative fit index; RMSEA, root mean square error of approximation; SRMR, standardized root mean square residual; CI, confidence interval.*

## Results

### Descriptive and Correlation Analysis

Table 3 shows that *teaching competence* presented moderately high average values, that for the ISC, the average values were higher for *bored* than for *satisfaction with school*, and that for disruptive behaviors, the average values were moderately low, oscillating between *low engagement or irresponsibility* and *poor self-management*, which presented the lowest average.

**TABLE 3:** Descriptives and correlations of the ECTS-PE, ISC, and CCDEF subscales.

Subscales	*M*	*SD*	1	2	3	4	5	6	7
(1) Teaching competence	5.36	1.16	−	0.19**	−0.13**	−0.19**	−0.08	−0.08	−0.09
(2) Satisfaction with school	2.80	0.97	0.23**	−	−0.40**	−0.06	0.01	−0.03	−0.03
(3) Boredom with school	3.03	1.04	−0.14**	−0.23**	−	0.30**	0.21**	0.26**	0.19**
(4) Low engagement or irresponsibility	2.00	0.90	−0.23**	0.06	0.21**	−	0.65**	0.53**	0.41**
(5) Fails to follow directions	1.65	0.94	−0.21**	0.10	0.11*	0.73**	−	0.62**	0.55**
(6) Distracts or disturbs others	1.49	0.99	−0.14*	0.08	0.20**	0.69**	0.75**	−	0.69**
(7) Poor self-management	1.42	0.83	−0.09	0.08	0.16**	0.68**	0.69**	0.83**	−

*p < 0.05; **p < 0.01; *M* = mean; *SD* = standard deviation. The upper diagonal corresponds to the girls. The lower diagonal corresponds to the boys.

The correlations show that *teaching competence* only presented positive, moderate, and statistically significant values for *satisfaction with school*. Disruptive behaviors presented high, positive and statistically significant correlations between the same CCDEF subscales although positive correlations with more moderate values were also found between the different disruptive and *boredom with school* subscales (see Table 3).

### Differences According to the Gender Variable

The differences were analyzed between the various subscales of teacher competence, school satisfaction and disruptive behaviors according to the gender variable. As shown in Table 4, the analyses indicate that there are statistically significant differences in the *boredom with school* and the four CCDEF subscales, and that, in all of them, the average values are higher for boys.

**TABLE 4:** Gender differences based on the ETCS-PE, ISC, and CCDEF subscales according to the mixed regression model.

				CI95%		Statistical tests adjusted mixed model			Effect size
						
							
	Gender	Adjusted average	Typical error	Lower	Higher	*F*	df	*p*	*d*
Teaching competence PE	Girl	5.43	0.06	5.31	5.54				
						2.49	756	0.115	0.115
	Boy	5.29	0.06	5.17	5.41				
Satisfaction with school	Girl	2.80	0.04	2.71	2.89				
						0.00	756	0.978	0.000
	Boy	2.79	0.05	2.70	2.89				
Boredom with school	Girl	2.89	0.05	2.79	2.99				
						17.36	756	0.000	0.304
	Boy	3.20	0.06	3.10	3.31				
Low engagement or irresponsibility	Girl	1.88	0.04	1.80	1.97				
						15.86	756	0.000	0.291
	Boy	2.14	0.05	2.05	2.23				
Fails to follow directions	Girl	1.53	0.04	1.45	1.61				
						20.06	756	0.000	0.327
	Boy	1.80	0.04	1.71	1.89				
Distracts or disturbs others	Girl	1.37	0.04	1.29	1.44				
						24.45	756	0.000	0.361
	Boy	1.65	0.04	1.56	1.73				
Poor self-management	Girl	1.25	0.04	1.17	1.33				
						42.74	756	0.000	0.477
	Boy	1.63	0.04	1.55	1.72				

*df, degrees of freedom; d, Cohen’s d.*

### Differences According to Teaching Competence

In order to check the differences in the *satisfaction with school* and the *disruptive behaviors* subscales, according to the three *teaching competence* groups (low, medium, and high), the analysis performed indicates that the *p*-value associated with the comparative statistical tests of marginal averages has been calculated and corrected for multiple comparisons using SIDAK (Table 5).

**TABLE 5:** Differences in teaching competence (ETCS-PE) based on the ISC and CCDEF subscales according to the mixed regression model.

				CI95%		Mixed model adjusted			SIDAK^b^	Effect size
							
	Groups ETCS-PE^a^	Adjusted average	Typical error	Lower	Higher	*F*	df	*p*	*p*	*d*
Satisfaction with school	1	2.38	0.15	2.08	2.68				3***	0.779
	2	2.69	0.04	2.61	2.77	16.77	755	0.000	3***	0.315
	3	3.02	0.05	2.92	3.13					

Boredom with school	1	3.39	0.18	3.03	3.75				3**	0.624
	2	3.14	0.05	3.04	3.23	10.74	755	0.000	3***	0.252
	3	2.81	0.06	2.68	2.93					

Low engagement or	1	2.35	0.16	2.04	2.66				3**	0.764
irresponsibility	2	2.12	0.04	2.04	2.20	16.13	755	0.000	3***	0.309
	3	1.76	0.05	1.66	1.87					

Fails to follow directions	1	1.84	0.15	1.55	2.13					
	2	1.74	0.04	1.67	1.82	9.42	755	0.000	3***	0.236
	3	1.48	0.05	1.38	1.58					

Distracts or disturbs others	1	1.78	0.14	1.51	2.06				3*	0.456
	2	1.54	0.04	1.47	1.61	5.74	755	0.003	3*	0.184
	3	1.38	0.05	1.29	1.47					

Poor self-management	1	1.73	0.15	1.44	2.02				3*	0.421
	2	1.47	0.04	1.39	1.54	4.96	755	0.007		
	3	1.32	0.05	1.22	1.47					

*p < 0.05, **p < 0.01, ***p < 0.001; d, Cohen’s d. ^*a*^Groups: 1 = low teaching competence (*n* = 31), 2 = medium teaching competence (*n* = 459), 3 = high teaching competence (*n* = 268); ^*b*^Comparison of marginal means statistical tests corrected by multiple comparisons using SIDAK. To simplify the presentation of data, only groups with statistically significant differences are displayed.

Table 5 shows that there are statistically significant differences in all the subscales studied. In the case of satisfaction with school, the highest averages correspond to the high teaching competence group, whereas for boredom with school and the four CCDEF subscales, the highest average values are presented by the low teaching competence group.

Regarding satisfaction with school and boredom with school, comparison tests show statistically significant differences between low and high teaching competence and between those of medium and high teaching competence, corrected using SIDAK (see Table 5). In the cases of disruptive behaviors, for low engagement or irresponsibility and fails to follow directions, statistically significant differences are notable between medium and high teaching competence; in the case of the Distracts or disturbs others subscale, statistically significant differences were found between high teaching competence and the other two groups, while in poor self-management, they were only found between low and high teaching competence.

## Discussion

This study set out two objectives: to understand the differences that exist in school satisfaction, disruptive behaviors and teaching competencies according to the gender of the students; and to analyze school satisfaction and disruptive behaviors based on teaching competence.

The results of this work relate teaching competence, satisfaction with school and inappropriate behaviors in the classroom. As in other studies (e.g., Kulinna et al., 2006; Cothran and Kulinna, 2007), the children presented higher levels of disruptive behavior. These results might be due to the boredom experienced by adolescents coming from a lack of attachment to social institutions and from disruptive behaviors at school (Feinberg et al., 2013; Granero-Gallegos et al., 2020). It is essential that students do not experience boredom in school, given that it is related to school violence, and this in turn can contribute to reduced academic performance, mental health and general well-being of the students (Huebner et al., 2014; Olweus and Breivik, 2014). In addition, boredom has been associated with high-risk behaviors such as drinking, drug use, joyriding and criminal activity (Yang and Yoh, 2005; Wegner and Flisher, 2009). Therefore, it is important that teachers work on their social skills with students and acquire sufficient competency as educators so that, amongst other things, both feel satisfied in classes (Allen et al., 2015; Trigueros and Navarro, 2019). Accordingly, this confirms Hypothesis 1.

If one looks at the mixed regression model, no significant differences were found in the teacher competence and school satisfaction variables based on gender. However, significant differences were found in the boredom with school, low engagement or irresponsibility, fails to follow directions, distracts or disturbs others and poor self-management variables, with boys presenting higher values than girls. These results are similar to those obtained in previous studies (e.g., Beaman et al., 2006; Kulinna et al., 2006; Cothran and Kulinna, 2007; Driessen, 2011), in which higher levels of disruptive behavior were also found in boys. They may be due to boys being more defiant with the teacher and more competitive with their peers, seeking to get the attention of the girls. In addition, it has been observed that males tend to engage in louder and more intentional behaviors to distract their peers in class (Glock and Kleen, 2017). Also, a possible cause for the increased level of negative behaviors has been linked to low emotional support from the teacher (Shin and Ryan, 2017). All this can be the basis for proposing more comprehensive teacher training, not only at the technical level, but also in the management of emotions, both in the initial training and in the continuous workplace training. In contrast, the girls presented more positive and less problematic behaviors, as was the case in other studies (e.g., Driessen, 2011). This may be because girls tend to demonstrate more introverted behavior, being uninvolved, shy and avoiding working as a group to give their opinion on a topic (Glock and Kleen, 2017). Therefore, this does not confirm Hypothesis 2 in its entirety.

The model analyzed based on teacher competencies found that when students perceived PE teachers as being competent, they felt more satisfied with the school, less bored and that their disruptive behavior level fell. Conversely, when students perceived their teachers as being incompetent, they became more bored and inappropriate behaviors increased. Similar results were found in the study by Baños et al. (2019), which was conducted in the same country as our work. These results suggest that the way teachers interact with their students affects classroom behavior (Ryan et al., 2015). This highlights the importance of PE teachers acquiring a great deal of skills to control and manage the sessions, creating a proactive environment among students, thus decreasing the likelihood of bad behaviors (Shin and Ryan, 2014; Fortuin et al., 2015). However, teachers reporting high levels of concern regarding how to effectively manage discipline issues in the classroom are common (Evertson and Weinstein, 2006; Tsouloupas et al., 2010) as they feel incompetent in the face of certain situations and this can be related to academic failure (Jurado-de-los-Santos and Tejada-Fernández, 2019). The inability to prevent and control student misbehavior is one of the main generators of teacher stress and anxiety, resulting in teachers burning out and increasing the likelihood of student truancy – with all the expenses that this involves for the educational system in terms of having to find substitute teachers (Tsouloupas et al., 2010; Ervasti et al., 2011). Therefore, this confirms Hypothesis 3.

PE teachers affirm that they find it more difficult to manage the boys’ behavior (Jackson and Smith, 2000). These higher management issues may be due to the fact that teachers assess the temperament and educational competence of boys more negatively than those of girls (Mullola et al., 2012) and that boys more frequently show emotional opposition behaviors than girls do (McClowry et al., 2013). These differential behaviors in students and the teachers’ perceptions are reflected in less intimate and more conflictive relationships between teachers and boys (Spilt et al., 2012). As a result, male students receive more reprimands (Beaman et al., 2006) than female students, making it harder to manage the boys’ behavior (McClowry et al., 2013). This implies less effective classroom management with respect to males, as research has emphasized the importance of positive relationships between the teachers and the students to promote good classroom management (Marzano and Marzano, 2003). Therefore, teacher training is needed to better support trust and good management in the classroom.

## Conclusion

The results obtained from this study identify males as having higher levels of inappropriate behaviors and the importance of students perceiving their teachers as being competent, that teachers have a command of the pedagogical content (Voss et al., 2011) and knowledge of classroom management techniques (Emmer and Stough, 2001) so that they can help reduce misbehavior in PE. Therefore, it is essential that adolescents perceive the PE teacher as competent, providing emotional support to his/her students, and that he/she continues to train in areas such as conflict resolution in the classroom, didactics and teacher pedagogy.

From this study, some recommendations can be made to bring, both to the classroom and to school. In general, the creation or strengthening of classrooms for school coexistence that improves the reflection, help, and accompaniment by other selected students can be recommended; it would be a program based on responsibility and without punishments or sanctions, and contribute to the resolution of conflicts in a positive way. By law, all educational centers must have a School Coexistence Plan, which must be implemented. More particularly, it is possible to focus on approaches that imply an enhancement of the motivation among students, especially in boys. Also, the enhancement of teaching competence in several topics (e.g., communication, work awareness, individual consideration of the student, problem-solving, social awareness, etc.), although the educational administration should supply teachers continuous training to improve social skills and capacity to solve conflicts among students.

### Limitations and Strengths

The notable strengths of this work are the sample size and the theme, which can contribute to remedying one of the main problems found on a day-by-day basis in secondary schools. However, despite the novelty and interest of the topic and the results provided in this study such as the relationship between teaching competence and disruptive behaviors, as well as the implications this might have at the pedagogical and teacher-training level, certain limitations should be taken into account. The sample is composed of secondary school students from a single autonomous region and, in addition, no probabilistic sample design was carried out, so the results cannot be generalized and the method used does not allow to deeper into the disruptive causes in the classroom. Further studies should be performed in which other research designs are proposed, such as experimental studies with intervention programs to reduce disruptive behaviors in the classroom, and which consider other variables related to teacher, or mixed quantitative and qualitative research designs could be proposed, focusing on all subjects, not just PE. Some of these studies could also include private schools and public schools located in different socioeconomic level areas. On the other hand, it would also be convenient to perform longitudinal researches, with various data collections, in which the effectiveness of coexistence programs is valued.

## Data Availability Statement

The datasets generated for this study are available on request to the corresponding author.

## Ethics Statement

The studies involving human participants were reviewed and approved by REF-45-20/01/2016. Written informed consent to participate in this study was provided by the participants’ legal guardian/next of kin.

## Author Contributions

AG-G, RB, and AB-E conceived the hypothesis of this study. RB, AB-E, and MM-M participated in data collection. RB and AG-G analyzed the data. AG-G, AB-E, and MM-M wrote the manuscript with the most significant input from AB-E. All authors contributed to data interpretation of statistical analysis and read and approved the final manuscript.

## Conflict of Interest

The authors declare that the research was conducted in the absence of any commercial or financial relationships that could be construed as a potential conflict of interest.
